# Traditional Chinese exercise (TCE) on pulmonary rehabilitation in patients with stable chronic obstructive pulmonary disease: Protocol for a systematic review and network meta-analysis

**DOI:** 10.1097/MD.0000000000016299

**Published:** 2019-07-05

**Authors:** Wenjiang Zheng, Meichen Li, Yu Hong, Fuqi Xie, Qian Yan, Zijing Peng, Huiting Huang, Huili Liao, Xiaohong Liu

**Affiliations:** a Guangzhou University of Chinese Medicine; b Clinical Medical College of Acupuncture Moxibustion and Rehabilitation, Guangzhou University of Chinese Medicine; c The First Affiliated Hospital of Guangzhou University of Chinese Medicine, Guangzhou, China.

**Keywords:** chronic obstructive pulmonary disease, network meta-analysis, protocol, systematic review, traditional Chinese exercise

## Abstract

**Background:**

Chronic obstructive pulmonary disease (COPD) has the characteristics of high incidence, mortality, disability rate, and heavy economic burden. Symptomatic measures such as anti-inflammatory, antispasmodic and anti-asthmatic are widely used in the treatment of COPD, and pulmonary rehabilitation has not been fully utilized. It is reported that up to 10 different kinds of Traditional Chinese exercises (TCEs) are often used for treating stable COPD. There are many randomized controlled trials (RCTs) and systematic reviews that have evaluated the efficacy of various TCEs for COPD. However, most of these studies were designed in comparison with conventional western medicine or health education. There are rarely studies to compare different TCEs head to head. Therefore, there remains uncertainty regarding the comparative efficacy among different TCEs. Thus, we plan to conduct a systematic review and Network meta-analysis (NMA) to compare the efficacy among 5 different TCEs and rank their benefits relative to each other. It is hoped that the findings of this study will facilitate the management and application of TCEs in the treatment of COPD.

**Methods:**

A systematic and comprehensive literature search will be performed from inception to April 2019 in both English and Chinese databases, involving Medline, Cochrane Library, Embase, China National Knowledge Infrastructure Database, Wanfang Database, China Biomedical Literature Database, and Chongqing VIP information. RCTs related to TCE in the treatment of COPD will be included. Quality of included trials will be assessed according to the risk of bias tool of Cochrane Handbook 5.1.0. The GRADE approach will be used to rate the certainty of the evidence of estimates derived from NMA. Data analysis will be conducted by using STATA 14.0.

**Results:**

This systematic review and NMA aims to summarize the direct and indirect evidence for different kinds of TCEs and to rank these TCEs. The findings of this NMA will be reported according to the PRISMA-NMA statement. The results of the NMA will be submitted to a peer-reviewed journal once completed.

**Conclusion:**

Using NMA, this study will provide an evidence profile which will be helpful to inform the selection of TCE for treating patients with COPD. The results will inform clinicians, bridge the evidence gaps, and identify promising TCE for future trials.

**PROSPERO registration number:**

PROSPERO CRD 42019132970.

## 1 Introduction

Chronic obstructive pulmonary disease (COPD) is a preventable and treatable lung disease characterized by incompletely reversible expiratory airflow limitation, which progressively develops and severely endangers human health. It is reported that COPD affects 380 million people worldwide, representing 12% of adults over 30 years of age, the prevalence of this disease is rapidly increasing, and it will be the third cause of mortality worldwide in 2020.^[1]^ At present, symptomatic measures such as anti-inflammatory, antispasmodic and anti-asthmatic are widely used in the treatment of COPD, and lung rehabilitation has not been fully utilized.^[2]^

Pulmonary rehabilitation, which includes strength and endurance training and educational, nutritional, and psychosocial support, improves symptoms and exercise tolerance. In the past few decades, pulmonary rehabilitation has become an integrated part of the management of individuals with moderate to very severe COPD. Exercise training is the cornerstone of pulmonary rehabilitation and mostly consists of aerobic exercises (stationary cycling, treadmill walking and/or ground walking) in combination with muscle strengthening exercises (resistance training and/or transcutaneous neuromuscular electrical stimulation). It is a comprehensive therapy to improve the physical and mental state of patients with chronic respiratory diseases. However, in the clinic, multiple members need to jointly to develop a good rehabilitation plan for patients, which make it difficult to carry out.

In contrast, Traditional Chinese exercises (TCEs) that involve human physical activity, breath expiration, and mind regulation are easy to master in a short time and have few physical demands.^[3]^ They also involve soft movements and coordination of respiration and movement. These exercises are easy and enjoyable to learn. Many studies have confirmed that long-term exercises like TaiChi positively affect physical function, exercise capacity, and psychological state and long-term practice of these exercises help in the treatment of chronic diseases. Regardless of previous exercise experience or aerobic capacity, the exercise intensity of TCEs is suited for persons of all ages. On the other hand, TCEs require no expensive equipment and can be performed either individually or in groups. Most existing systematic reviews have focused on specific forms of TCEs, such as TaiChi, Ba Duan Jin, or Yi Jin Jing.

Up to 10 different kinds of TCEs are reportedly often used for treating stable COPD; however, there are very few head-to-head comparative trials to determine the relative efficacy between different TCEs. Due to the fact that various TCEs are used in the clinic, it is difficult for clinicians to choose the optimal TCE for patients with COPD. We will conduct a Network meta-analysis (NMA) to compare the efficacy of different TCEs, including direct and indirect comparisons between TCEs. It is hoped that this NMA could provide the best currently available evidence base to guide the choice regarding TCE treatment for patients with COPD.

## 2 Methods

### 2.1 Study registering and reporting

The study protocol has been registered on PROSPERO (International Prospective Register of Systematic Reviews) (CRD42019132970). This protocol is developed in accordance with the Preferred Reporting Items for Systematic Reviews and Meta-analyses Protocols (PRISMA-P).^[4]^ Any protocol modifications made during the performing of the review will be recorded in the publication of the final report. The PRISMA Extension Statement is used to ensure all aspects of methods and findings are reported.^[5]^

### 2.2 Eligibility criteria

The PICOS (Population-Intervention-Comparators-Outcomes-Study design) framework was adopted as the eligibility criteria for the review as follows.

#### 2.2.1 Study design

Regardless of whether blinding is used or not, Studies were eligible for inclusion in this review if they satisfied the following criteria:

1. randomized controlled trial;
2. included COPD patients without restrictions in sex, age, and race, and the ratio of forced expiratory volume in one second (FEV_1_) to forced vital capacity (FVC) was less than 70% or FEV_1_ was less than 80% of predicted values according to the Global Initiative for Chronic Obstructive Lung Disease (GOLD) criteria;
3. TCEs intervention lasted for more than 12 weeks;
4. and outcome measures included FEV1, FEV_1_%, FEV_1_/FVC%, AE, 6MWD, HAMA, HAMD, SGRQ, CRQ, or CAT.

Studies that comprised patients who were not recently diagnosed with COPD or enrolled patients with acute exacerbation were excluded.

The following study designs or publication types will be excluded:

1. non-clinical research literature, such as animal experiments, reviews or case reports;
2. duplicate publications;
3. literature with incomplete data, the study of chaos and
4. studies which lack primary outcome measures.

If multiple intervention data can be obtained, the trails can be adopted. If data for comparison of multiple interventions cannot be directly obtained, we will try e-mailing the corresponding author to obtain the original data. If the data cannot be obtained, the trails will also be excluded.

#### 2.2.2 Population

According to the GOLD diagnostic criteria,^[6]^ RCTs with a definite diagnosis of COPD will be taken into consideration. The stable phase of COPD generally refers to without acute exacerbation within 4 weeks. There will be no limits on age, gender, race, or nationality.

#### 2.2.3 Interventions/comparators

TCEs are traditional Chinese forms of conditioning exercises derived from martial arts. When practiced correctly, it is thought to strengthen the body's vital energy and enhance the passage of this energy throughout the body to confer its health-promoting effects. They are characterized by posture alignment, weight shifting, breath balance, and circular movements that incorporate elements of muscle endurance and strengthening, balance, relaxation, and breathing control. It is reported that TCEs are often used for treating stable COPD and most existing systematic reviews have focused on specific forms of TCEs, such as TaiChi, Ba Duan Jin, or Yi Jin Jing.

The experimental group used traditional exercise therapy (such as TaiChi, Ba Duan Jin, Wu Qin Xi, Liu Zi Jue, or Yi Jin Jing) or combined with other interventions. The control group used conventional therapy (such as conventional medication, routine health guidance, etc.).

#### 2.2.4 Outcome measures

1. Pulmonary function: FEV_1_, FEV_1_%, FEV_1_/FVC%;
2. The incidence of Acute Exacerbations (AE);
3. Exercise endurance: 6-minutes walking distance (6MWD);
4. Emotional change: Hamilton Anxiety Scale (HAMA) and Hamilton Depression Scale (HAMD);
5. Health-related quality: St. George's respiratory questionnaire (SGRQ) and Chronic respiratory questionnaire (CRQ);
6. Health status: COPD assessment test (CAT).

### 2.3 Data sources and search strategy

The literature search will be conducted in three English databases (Medline, Cochrane Library, Embase) and four Chinese databases (China National Knowledge Infrastructure Database, Wanfang Database, China Biomedical Literature Database, and Chongqing VIP information) from inception to April 2019. A separate search for systematic reviews will be performed to compare the included studies from existing reviews against those retrieved from the current RCT searching. We will also undertake a targeted gray literature search on Clinical Trials.gov and the International Clinical Trials Registry Platform search portal to identify in-progress and completed trials. In addition, the search will include Google Scholar, Web of Science, and Baidu Scholar to identify trial protocols and other information. Further studies will be identified by examining the reference lists of all included studies.

We used the following combined text and Medical Subject Headings (MeSH) terms:“TaiChi”, “Ba Duan Jin”, ”Yi Jin Jing“, ”Liu Zi Jue“, ”Wu Qin Xi”, “COPD”, “chronic obstructive pulmonary disease”, “pulmonary disease” and “exercise capacity”. Take TaiChi as an example, Search terms used in PubMed were (“taiji” [MeSH] OR tai ji OR tai chi OR taijiquan OR qigong OR traditional Chinese exercise) AND (“lung disease, obstructive” [MeSH] OR “pulmonary disease, chronic obstructive” [MeSH] OR COPD OR chronic obstructive lung disease OR bronchiti∗, chronic∗ OR emphysema∗) AND (controlled clinical trial [pt] OR randomized controlled trial [pt] OR randomized [tiab] OR placebo [tiab] OR group [tiab] OR randomly [tiab]). To ensure a thorough search of the literature, we also hand-searched references of key articles that were published in English and in Chinese.

### 2.4 Study selection and data extraction

Records downloaded from eight databases will be managed by EndNote X9. Data were independently extracted by two researchers (Meichen Li, Fuqi Xie). Independently screen the included studies, extract data, evaluate the quality of included studies and cross-check with each other according to the established selection criteria. Disagreements will be resolved by discussion or consultation with a third author (Yu Hong). First, the preliminary screening will be performed by reading the title and abstract of the obtained literature. Studies that fail to meet the eligibility criteria will be excluded. Then full text of the articles will be retrieved to further determine whether they are included. Microsoft Excel will be used to extract data and collect relevant information. We extracted and recorded the first author's name, year of publication, study design, intervention, and control group information, sample size, duration of intervention, and outcomes, including FEV_1_, FEV_1_%, FEV_1_/FVC%, AE, 6MWD, SGRQ, CRQ, HAMA, HAMD and CAT results. We contacted the corresponding authors for additional information if necessary.

### 2.5 Quality assessment

The Risk of Bias Tool (ROB) in Cochrane Handbook (5.1.0) will be used to assess the methodological quality of included studies by two independent reviewers (Zijing Peng, Qian Yan). Disagreements will be resolved by discussion with a third reviewer (Xiaohong Liu). Seven items are included in the ROB: random sequence generation, allocation concealment, blinding of participants and personnel, blinding of outcome assessment, incomplete outcome data, selective reporting and other sources of bias. The judgment of each item is divided into three levels: low risk of bias, high risk of bias and unclear risk of bias.

The complete assessment procedures are as follows:^[7]^

1. offer direct and indirect effect estimates;
2. assess the quality of direct and indirect estimates;
3. present the results of the NMA and
4. assess the quality of the NMA effect estimates.

We will assess the certainty of evidence contributing to network estimates of the primary outcome through The Grading of Recommendations Assessment, Development and Evaluation (GRADE) system. Based on five key areas (bias, indirection, inconsistency, imprecision, and risk of publication bias), the quality of evidence is divided into one of four levels: high, medium, low, and very low.

### 2.6 Dealing with missing data

We will initially contact both senior and/or corresponding author to obtain any missing data. If no one responds, the following approaches will be used to estimate the missing data. Instead of providing the mean and Standard Deviation, the number of responding patients employing a validated imputation method will be calculated for studies failing to report the patients’ numbers after treatment. We will also try to estimate from graphs if possible. The reason for the exclusion of the available data will be reported.^[8]^

### 2.7 Statistical analysis

(1) The ROB in Cochrane Handbook 5.1.0 will be used to assess the methodological quality of the included studies. Weighted Mean Difference (WMD) or Standardized Mean Difference (SMD) with 95% confidence interval (95%CI) will be calculated for continuous data (FEV_1,_ FEV1%, FEV_1_/FVC%, AE, 6MWD, HAMA, HAMD, SGRQ, CRQ, and CAT).

The heterogeneity test will be performed by using STATA 14.0 software. If *I*^*2*^ ≤ 50% and *P* ≥ .05, it suggests that heterogeneity is not important and the Mantel-Haenszel fixed model will be employed for meta-analysis. If *I*^*2*^>50% and *P* < .05, it manifests that heterogeneity needs to be analyzed. Subgroup analysis, meta-regression or descriptive analysis will be used for heterogeneity analysis.^[9]^

The NMA will be conducted using the network command in STATA to rank probabilities of treatments. Then perform the heterogeneity test and the inconsistency test. We will employ the Inconsistency Factor (IF) to evaluate heterogeneity among the included studies if a closed loop exists. If the 95% CIs of the IF values are truncated at zero, it indicates that direct and indirect evidence is in agreement. A comparison-adjusted funnel plot will be conducted to assess the presence of a small-study effect.

### 2.8 Patient and public involvement

This part is not covered in this study.

## 3 Discussion

At present, various therapies for COPD can alleviate symptoms to a certain extent and reduce the acute onset of COPD, but there is no effective method for eradicating COPD. Throughout the clinical practice guidelines at home and abroad, the current research focuses on how to effectively control the condition of COPD, slow down the development of the disease and the deterioration of pulmonary function, and improve the quality of life of patients. Pulmonary rehabilitation is recommended as one of the most basic comprehensive therapies for patients with stable COPD. It incorporates strength and endurance training and educational and nutritional and psychosocial support and can improve cardiovascular fitness, physical activity levels, and symptoms in patients with COPD. Exercise training is a major component of the Psychological Rehabilitation Program (PRP), which is widely used in the clinical treatment of patients with COPD.

Despite these benefits, many problems remain unresolved. First, considered globally, fewer than 5% of eligible patients receive pulmonary rehabilitation,^[10]^ in part due to funding or reimbursement issues;^[11]^ Second, conventional exercise training requires gym equipment and space in which to house them, which entails the creation of a central location; travel distance to the pulmonary rehabilitation facility has been identified as a barrier to participation. Third, since the positive effects of pulmonary rehabilitation tend to diminish over time one would expect them to be extended by continued exercise.^[12–16]^

In this respect, TCEs may offer a number of attractions compared with conventional exercise-based pulmonary rehabilitation and may, therefore, facilitate exercise training for some individuals with COPD. TCEs might very well be cheaper, particularly in countries with a large practitioner base, which makes it a low-cost, easily accessible alternative. Moreover, it requires no equipment and minimal space. Because it requires no apparatus, TCEs may be more attractive for maintenance therapy, after an original course of either TCEs or standard exercise-based pulmonary rehabilitation, than continued western-style exercise.^[17,18]^

Numerous studies have confirmed that pulmonary dysfunction reduces exercise capacity in COPD patients; moreover, the decreased exercise capacity further lowers their quality of life,^[19]^ which makes it easier for patients to be prone to negative emotions such as anxiety and depression. Spirometry is the reference standard for diagnosing and assessing the severity of COPD.^[20]^ The incidence of AE is the most frequent reason for hospital admissions and death and negatively influence the quality of life and prognosis in patients with COPD. The 6MWD is a valid indicator for evaluating the exercise capacity of COPD patients.^[21,22]^ The HAMA and HAMD is a validated and reliable indicator for evaluating the emotional change of COPD patients.^[23]^ Quality of life in COPD patients is usually assessed by the SGRQ or CRQ.^[24]^ CAT is an important indicator for evaluating the health status of COPD patients. We will conduct a NMA to compare the efficacy of different TCEs, including direct and indirect comparisons between TCEs to compare the efficacy among 5 different TCEs and rank their benefits relative to each other. It is hoped that the findings of this study will facilitate the management and application of TCEs in the treatment of COPD.

Three issues will need specific attention in this study. First, we have found that randomization was not designed rigorously in most studies published in Chinese journals based on our experience. Predictably, most of the randomized controlled trials included in this study will be rated as low-level quality.^[5]^ Second, this study is based on a literature analysis, which is not a direct head to head comparative study. The relative efficacy between TCEs will be estimated from a common comparator indirectly using a NMA. The presence of heterogeneity is an inherent problem in a meta-analysis, which would affect the estimate. It results from the diversity in clinical and methodological characteristics, and variations between studies.^[9]^ Third, as an emerging statistical method, some limitations remain in the use of NMA. A high quality NMA depends on three conditions-network connectivity, the similarity of trials with respect to study design and populations, and network consistency.^[25]^ Thus, we cannot guarantee that the relative efficacy of the difference among TCEs is the true value as it is in practice. Consequently, further head to head comparisons are required to confirm the results.

As this study is secondary research based on literature, ethics approval and patient consent are not necessary. This protocol is designed in accordance with guidelines for NMA protocols. It will be conducted and reported according to the PRISMA extension statement for NMA. The results of this NMA will be submitted to a peer-reviewed journal once it is completed.

## Author contributions

**Conceptualization:** Wenjiang Zheng, Meichen Li, Xiaohong Liu.

**Data curation:** Meichen Li, Yu Hong, Fuqi Xie, Zijing Peng, Huiting Huang.

**Formal analysis:** Yu Hong, Qian Yan, Zijing Peng.

**Funding acquisition:** Qian Yan, Huiting Huang, Xiaohong Liu.

**Investigation:** Wenjiang Zheng, Qian Yan.

**Methodology:** Meichen Li.

**Project administration:** Fuqi Xie.

**Resources:** Fuqi Xie, Zijing Peng, Huili Liao, Xiaohong Liu.

**Software:** Wenjiang Zheng, Huili Liao.

**Supervision:** Huiting Huang, Huili Liao, Xiaohong Liu.

**Validation:** Xiaohong Liu.

**Visualization:** Qian Yan.

**Writing – original draft:** Wenjiang Zheng, Meichen Li, Yu Hong, Xiaohong Liu.

**Writing – review & editing:** Wenjiang Zheng, Xiaohong Liu.

Xiaohong Liu orcid: 0000-0003-4795-3039.
